# Aflatoxin B1 triggers ferroptosis via inhibiting the NRF2 signaling pathway in duck granulosa cells

**DOI:** 10.1016/j.psj.2025.105993

**Published:** 2025-10-24

**Authors:** Yaru Chen, Lei Wang, Ming Fu, Tao Huang, Hao Zhang, Jie Shen, Ailuan Pan, Zhenhua Liang, Jing Sun, Jinping Du, Jinsong Pi, Yan Wu

**Affiliations:** a Institute of Animal Science and Veterinary Medicine, Hubei Academy of Agricultural Sciences, Wuhan 430064, China; b Hubei Key Laboratory of Animal Embryo Engineering and Molecular Breeding, Wuhan 430064, China; c Wuhan Green Giant Agriculture & Animal Husbandry Company Limited, Wuhan 432200, China

**Keywords:** Selenomethionine, AFB1, Ferroptosis, NRF2, Granulosa cell

## Abstract

Aflatoxin B1 (**AFB1**) is an unavoidable food and environmental contaminant that exerts toxicity on female reproductive system, poses major threats to livestock and poultry breeding. However, the underlying mechanisms of AFB1-induced reproductive toxicity and effective interventions remain unclear. We observed that AFB1 exposure significantly suppressed granulosa cell growth and cell viability in ducks. Further analysis revealed that AFB1 exposure triggered excessive oxidative stress in duck granulosa cells. Additionally, AFB1 exposure induced lipid peroxidation, mitochondrial dysfunction and ferroptosis, which were alleviated by Ferrostatin-1. Transcriptomic analysis revealed the suppression of the NRF2 signaling pathway in AFB1 exposure. Meanwhile, NRF2 inhibitor (**ML385**) abolished the protective effect of Ferrostatin-1 and exacerbated ferroptosis, suggesting that NRF2 plays a protective role against AFB1-induced cytotoxicity. Moreover, seleniummethionine (**Se-Met**), an NRF2 pathway activator, effectively alleviated AFB1-induced granulosa cell damage. This study elucidates the protective mechanism of the NRF2 signaling pathway against AFB1-induced ferroptosis and highlights the potential of Se-Met as a therapeutic strategy for alleviating AFB1-induced reproductive dysfunction in poultry.

## Introduction

The ovary is a critical reproductive organ that orchestrates follicular development and maturation, hormonal secretion and reproductive cyclicity (Migale et al., 2024). Dysregulated ovarian development or functional impairment can severely compromise poultry reproductive potential and egg production (Li et al., 2022). Ovarian folliculogenesis is a remarkably complex, well-orchestrated process that relies on the synchronization communication between oocytes and surrounding granulosa cells (Gao et al., 2024). Follicular growth and development are significantly regulated through programmed cell death mechanisms in granulosa cells including autophagy, apoptosis, and necrosis. Indeed, most follicles undergo atresia during development, but less than 5 % follicles transition from the primordial follicle to the mature follicle and ultimately ovulate (Jo et al., 2025).

Ferroptosis, a newly identified form of programmed cell death, is characterized by iron‐dependent accumulation of lipid hydroperoxides and reactive oxygen species (**ROS**) (Dixon and Olzmann, 2024). Distinct from other forms of regulated cell death (RCD), ferroptosis exhibits abnormal mitochondrial morphology, excessive oxidative stress, and accumulation of lipid peroxides and ROS. Multiple studies have established a significant association between ferroptosis and follicular development (Liu et al., 2023b). For instance, the ferroptosis inhibitor (Ferrostatin-1, **Fer-1**) treatment alleviates hyperandrogenism and ovulatory dysfunction in the polycystic ovary syndrome (PCOS) rat models (Li et al., 2024b). BECN1 deletion in mice triggers oocyte ferroptosis, leading to premature ovarian insufficiency and fertility decline (Wang et al., 2022). Furthermore, polyvinyl chloride (PVC) microplastics promotes ferroptosis in ovarian, which subsequently disrupts follicular development and reduces egg production in ducks (Ali et al., 2024). Collectively, these findings reveal that ferroptosis plays a crucial role in regulating reproductive function.

Aflatoxins (**AFs**) are a group of mycotoxins predominantly produced by Aspergillus flavus and Aspergillus parasiticus through secondary metabolic pathways (Guo et al., 2021). AFs, growing rapidly in environments with high temperatures and high humidity, are widely present in soil, cereal crops and silage. AFs are highly stable compounds that are difficult to remove from contaminated foodstuffs and feed. Consequently, they have been included in the Rapid Alert System for Food and Feed (Malekinezhad et al., 2021). Notably, Aflatoxin B1 (**AFB1**) is the most toxic among these known aflatoxin derivatives, and AFB1 exposure can lead to various negative effects in animal and human health (Cheng et al., 2023a). Numerous studies have demonstrated that AFB1 exerts hepatotoxicity, nephrotoxicity, immunotoxicity, carcinogenicity and reproductive toxicity, and the toxicity mechanisms of AFB1 correlates with oxidative stress, cell death, inflammatory responses and several signaling pathways (Cheng et al., 2023b).

Aflatoxins exposure has been shown to reduce reproductive performance in livestock and poultry (Li et al., 2024a). Multiple studies have indicated that AFB1 could impair follicular development by disrupting redox homeostasis, cell growth and survival, and steroid hormone synthesis (Cheng et al., 2019). Recently, it has been suggested that AFB1-induced liver toxicity via microbiota-gut-liver axis-mediated ferroptosis in chicken (Huang et al., 2023). Furthermore, AFB1 exposure impairs blood-brain barrier function by disrupting redox homeostasis and activating ferroptosis in mice (Lin et al., 2025). However, the potential role of ferroptosis in AFB1-induced follicular developmental impairment remains poorly understood.

In this study, we systematically investigated the toxic effects of AFB1 on follicular development in ducks and its potential mechanisms using in *vitro* cultured duck ovarian and granulosa cell models. Our findings indicated that AFB1 treatment induces oxidative stress, lipid peroxidation, mitochondrial dysfunction and iron deposition, but these effects were alleviated by ferroptosis inhibitors (Ferrostatin-1, Fer-1) treatment. Furthermore, the NRF2 signaling pathway was found to protect granulosa cells and ovarian from AFB1-induced oxidative stress and ferroptosis in ducks. These findings provide a new theoretical basis for the reproductive toxicity mechanism of AFB1 and propose effective intervention strategies for protecting follicular development in poultry.

## Methods and materials

### Chemicals and reagents

AFB1 (≥99.8 % purity, 1162-65-8) was purchased from Pribolab (Qingdao, China). Ferrostatin-1 (99.71 %, HY-100579) was purchased from MCE (New Jersey, USA). ML385 (99.92 %, S8790) was purchased from Selleck (Shanghai, China). Se-Met (99.86 %, HY-B1000) was purchased from Selleck (New Jersey, USA).

### Cell isolation and culture

Hierarchical follicles were isolated from duck ovaries as previously described (Li et al., 2025a). After removing connective tissue, the granulosa layer was separated post-yolk extrusion, minced, and digested in collagenase type II. The digested mixture was filtered through 70 μm strainers to isolate granulosa cells. Cells were cultured in M199 (11150059, Gibco, Thermo Fisher Scientific, Waltham, MA, USA) supplemented with 10 % fetal bovine serum (10099141C, Gibco, Thermo Fisher Scientific, Waltham, MA, USA) and 1 % penicillin/streptomycin (11360, Invitrogen, Carlsbad, CA) in a humidified atmosphere (5 % CO2, 37°C).

The specific model establishment is as follows:

**AFB1 Exposure Granulosa Cells Model Establishment:** Control group received 0 μM AFB1; AFB1 exposure group: granulosa cells were treated with different concentrations of AFB1 (0.625, 1.25, 2.5, 5, 10 μM).
**Fer-1 Intervention on AFB1-Exposed Granulosa Cells Model Establishment:** Fer-1 intervention group: granulosa cells were treated with Fer-1 (0.5 μM); Fer-1 and AFB1 intervention group: granulosa cells were treated with Fer-1 (0.5 μM) and AFB1 (5 μM); Fer-1, AFB1 and ML385 intervention group: granulosa cells were treated with Fer-1 (0.5 μM), AFB1 (5 μM) and ML385 (3 μM).
**Se-Met Intervention on AFB1-Exposed Granulosa Cells Model Establishment:** Se-Met intervention group: granulosa cells were treated with different concentrations of Se-Met (0.1, 0.3 0.5 μM); Se-Met and AFB1 intervention group: granulosa cells were treated with Se-Met (0.5 μM) and AFB1 (5 μM); Se-Met, AFB1 and ML385 intervention group: granulosa cells were treated with Se-Met (0.5 μM), AFB1 (5 μM) and ML385 (3 μM).

### Cell viability assay

Cell viability was quantified using the CCK-8 assay (CA1210, Solarbio, Beijing, China). Briefly, 10 μL of CCK-8 reagent was added to each well and incubated at 37°C for 2-4 hours before measuring the absorbance using a microplate reader.

### Calcein-AM/PI staining

The co-staining of live and dead cells was performed using the Calcein-AM/PI Double Stain Kit (40747ES76, Yeasen, Shanghai, China). Granulosa cells were washed with PBS and incubated with Calcein-AM (2 μM) and PI (4 μg/mL) for 20 min at 37°C in dark. Fluorescence images were captured immediately using a fluorescence microscope: viable cells (green, Calcein-AM,) and dead cells (red, PI). The ratio of viable cells (Calcein-AM⁺) to total cells (Calcein-AM⁺ + PI⁺) was quantified using ImageJ software.

### Reactive oxygens species (ROS) content detection

The ROS levels of granulosa cells were quantified using the Reactive Oxygen Species Assay Kit (50101ES01, Yeasen, Shanghai, China). First, granulosa cells were harvested and incubated with 10 μM DCFH-DA for 2 h at 37°C in dark, and then washed three times and photographed with a fluorescence microscope. ROS levels were analyzed using Image J software.

### Measurement of GSH, GSSG, MDA, SOD, and CAT levels

The levels of glutathione (**GSH**, A006-2-1), oxidized glutathione (**GSSG**, A005-1-1), malondialdehyde (**MDA**, A003-1-2), superoxide dismutase (**SOD**, A001-3-2), and catalase (**CAT**, A007-1-1) were quantified using the commercially available kits (Nanjing Jiancheng Bioengineering Institute, China). All procedures were performed strictly following the manufacturer’s protocols.

### Assessment of mitochondrial membrane potential

Mitochondrial membrane potential (ΔΨm) was measured using JC-1 Assay Kit (C2006, Beyotime Biotechnology, Shanghai, China). The granulosa cells were washed with PBS and incubated with JC-1 working solution for 20 min at 37°C in dark. JC-1 exhibits potential-dependent accumulation in mitochondria, forming red fluorescent aggregates at high ΔΨm and green fluorescent monomers at low ΔΨm. The ΔΨm was quantified as the ratio of red (aggregate) to green (monomer) fluorescence intensity. Fluorescence was visualized by confocal microscopy and quantified (red/green ratio) using Image J software.

### Lipid peroxidation assay

Lipid peroxidation was quantified using BODIPY™ 581/591 C11 Kit (D3861, Thermo Fisher Scientific, Waltham, MA, USA). The granulosa cells were incubated with 2.5 μM C11-BODIPY (581/591) for 15 min at 37°C in dark. The granulosa cells were photographed with a confocal microscope and fluorescence intensity was quantified using Image J software. The level of lipid peroxidation was determined by calculating the ratio of oxidized (Green) to reduced (Red) fluorescence intensity.

### Mitochondrial permeability transition pore (MPTP) analyses

Mitochondrial permeability transition pore (**MPTP**) was assessed using the MPTP Assay Kit (C2009S, Beyotime Biotechnology, Shanghai, China). Briefly, differentiated brown and beige adipocytes were washed with PBS and incubated with 1 μM calcein-AM for 15 min at 37°C, followed by quenching with 1 mM CoCl₂ for 1 h. Subsequently, granulosa cells were photographed with a confocal microscope and fluorescence intensity was quantified using Image J software.

### Transmission electronic microscopy

For tissue samples, ovarian tissues were fixed in 2.5 % glutaraldehyde/0.1 M phosphate buffer (pH 7.4) for 30 min at 4°C, followed by post-fixation in 1 % osmium tetroxide in the same buffer for 30 min. After en bloc staining with 1 % uranyl acetate for 30 min, samples were dehydrated through a graded ethanol series (50 %, 70 %, 85 %, 95 %, and 2 × 100 %), then embedded in Eponate 12 resin (18005, Ted Pella, USA). Ultrathin sections (70 nm) were cut using a Leica EM UC6 ultramicrotome, double-stained with 2 % uranyl acetate and Reynold’s lead citrate. For cell sample, granulosa cells were harvested and fixed with 2.5 % glutaraldehyde. The granulosa cells were treated with osmic acid, ethanol, acetone, and other steps as described above. Ultrastructural analysis was performed using an FEI Tecnai G2 Spirit BioTWIN transmission electron microscope operating at 80 kV.

### FerroOrange staining

Intracellular ferrous iron (Fe²⁺) levels were quantified using the FerroOrange probe (F374, Dojindo Laboratories, Kumamoto, Japan). The granulosa cells were incubated with FerroOrange (1:2000 dilution in culture medium) at 37°C for 30 min in dark. The granulosa cells were photographed with a confocal microscope and fluorescence intensity was quantified using Image J software.

### Prussian blue staining

Intracellular iron distribution was assessed using Prussian blue staining (G1429, Solarbio, Shanghai, China). Ovarian sections and granulosa cells were fixed with 4 % paraformaldehyde for 15 min, then incubated with 10 % potassium ferrocyanide at 37°C for 30 min. After washing with PBS three times, nuclei were counterstained with Nuclear Fast Red for 10 min. Cells exhibiting brown granules were identified as Prussian blue-positive. The ovarian sections and granulosa cells were visualized and photographed by light microscopy, and then quantified using Image J software.

### Transcriptome analysis

Transcriptome sequencing was performed as previously reported with modifications (Xu et al., 2019). Granulosa cells isolated from duck ovarian follicles were divided into control and AFB1-treated groups (*n* = 4 per group). Total RNA was isolated using TRIzol reagent (Invitrogen, Carlsbad, USA) following the manufacturer's protocol. RNA integrity was verified by agarose gel electrophoresis and Nanodrop quantification before being processed for library construction.

All sequencing procedures were conducted by Lianchuan Biotechnology (Hangzhou, China), including: (1) polyA-selected mRNA enrichment, (2) cDNA library preparation using the Illumina TruSeq platform, and (3) paired-end 150 bp sequencing on an Illumina NovaSeq 6000 system. Raw read quality assessment using FastQC (v0.11.9) preceded differential expression analysis (|log_2_FC|>1, adjusted *p*-value<0.05). Functional annotation of DEGs was conducted through GO (Gene Ontology) and KEGG pathway enrichment analyses using clusterProfiler package (v4.0.5).

### RNA extraction and real-time quantitative PCR (RT-qPCR)

Total RNA was isolated from duck granulosa cells using TRIzol reagent (Invitrogen), with quality (A260/280 ratio >1.8) and concentration verified by NanoDrop 2000. RT-qPCR was used to examine the mRNA gene expression of NRF2 signaling pathway-related genes such as nuclear factor erythroid 2-related factor 2 (**NRF2**), heme oxygenase-1 (**HO-1**), glutathione peroxidase 4 (**GPX4**), solute carrier family 7 member 11 (**SLC7A11**), and ferritin heavy chain 1 (**FTH1**). Reverse transcription was performed using RevertAid RT Kit, followed by quantitative PCR with iTaq Universal SYBR Green Supermix on a CFX384 system. Gene expression was normalized to β-actin and calculated via the 2^-ΔΔCt^ method, and the related primers are listed in Table S1.

### Immunofluorescence

For immunofluorescence, granulosa cells were fixed with 4 % paraformaldehyde for 15 min, permeabilized with 0.1 % Triton X-100 in PBS for 5–10 min, and blocked with 10 % goat serum (v/v) or 5 % bovine serum albumin (BSA, w/v) in PBS for 1 h. The cells were then incubated overnight at 4°C with primary antibodies diluted in blocking buffer. After washing with PBS, the samples were incubated with appropriate fluorophore-conjugated secondary antibodies for 1 h in dark. Nuclei were counterstained with 4′,6-diamidino-2-phenylindole for 5 min. The cells were washed with PBS and mounted with antifade mounting medium. Fluorescence images were captured using a confocal microscopy. The information of antibodies was displayed in Table S2.

### Ovarian tissue culture

Fresh ovarian tissues were immediately transferred into ice-cold M199 medium (11150059, Gibco, Thermo Fisher Scientific, USA) supplemented with 1 % penicillin-streptomycin (15140122, Gibco, Thermo Fisher Scientifi, Waltham, MA, USA). After removing surface connective tissue under sterile conditions, intact follicles were sectioned into 1-2 mm^3^ explants using micro-scissors while preserving tissue architecture. The explants were placed on 0.4 μm cell culture inserts (353090, Corning Incorporated, USA) in 6-well plates with the basal medium consisting of M199 containing 10 % FBS (10099141C, Gibco, Thermo Fisher Scientific, Waltham, MA, USA), 1 % ITS (41400045, Gibco, Thermo Fisher Scientific, Waltham, MA, USA), and 50 ng/mL amphiregulin (989-AR-050, R&D, MN, USA). Tissues were maintained at 37°C in a humidified 5 % CO₂ atmosphere for up to 72 h, with medium changes every 24 h.

### Histology hematoxylin-eosin (H&E) staining

Ovarian tissues were fixed in 4 % paraformaldehyde (P1110, Solarbio, Beijing, China) for 24 h at 4°C, followed by dehydration through graded ethanol (70 %−100 %), xylene clearing, and paraffin embedding. Serial 5-μm sections were cut using a rotary microtome, mounted on poly-lysine-coated slides, and dried at 60°C for 2 h. After deparaffinization with xylene and rehydration through graded ethanol, sections were stained with Mayer's hematoxylin for 5 min, differentiated in 1 % acid alcohol, and blued in 0.2 % ammonia water. Counterstaining was performed with eosin for 3 min. Sections were dehydrated, cleared, and mounted with neutral balsam. Histological evaluation was conducted under a light microscope with image capture using Image J software.

### Statistical analysis

Data are expressed as means ± standard deviation (SD), with each experiment independently repeated at least three times for quantification. For comparisons between two groups, a two-tailed Student’s t-test was applied. Statistical significance was defined as * *p* < 0.05 and ** *p* < 0.01. Graghpad Prism 8.0 was used to draw Fig.s. The graphical abstract was made by Figdraw.

## Results

### AFB1 suppresses cell growth and induces oxidative stress

**Fig. 1:**
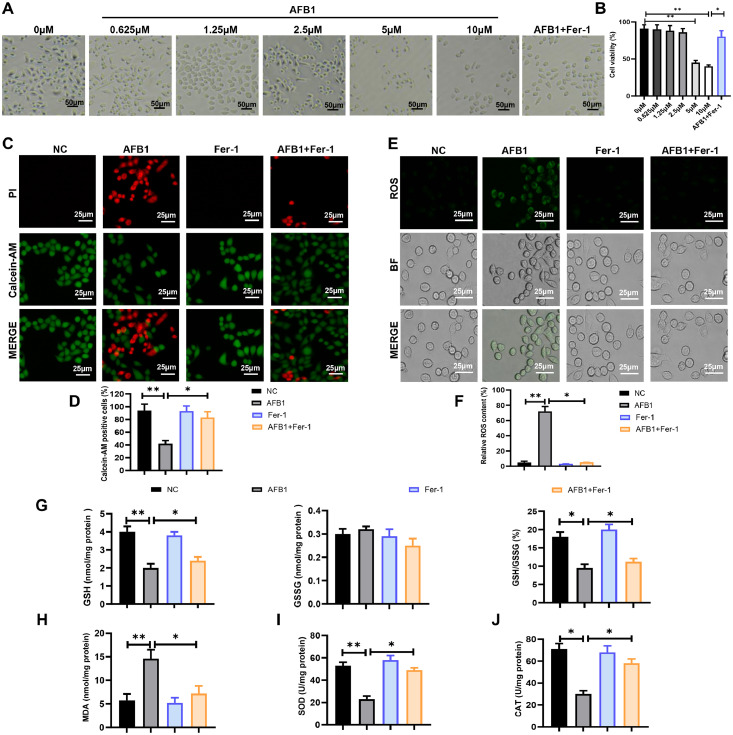
**AFB1 induces excessive oxidative stress in duck granulosa cells.** (A, B) Representative images and cell viability of duck granulosa cells treated with different concentrations of AFB1 and Fer-1. (C, D) Representative micrographs and quantification analysis of Calcein–AM/PI double staining in duck granulosa cells treated with AFB1 and Fer-1. (E, F) Fluorescence intensity and quantification analysis of ROS in duck granulosa cells treated with AFB1 and Fer-1. (G) The content of GSH, GSSG and the ratio of GSH/GSSG in duck granulosa cells treated with AFB1 and Fer-1. (H-J) The content of MDA (H), SOD (I), CAT (J) in duck granulosa cells treated with AFB1 and Fer-1. All data are mean ± SEM; *p < 0.05, **p < 0.01.

In this study, duck granulosa cells were treated with different concentrations of AFB1 (0, 0.625, 1.25, 2.5, 5, 10 μM) to assess effects on cell viability. The results showed that only 5 and 10 μM concentration of AFB1 significantly reduced the granulosa cell viability, thus we chose 5 μM AFB1 for subsequent experiments (Fig. 1A and B). Notably, Fer-1 co-treatment effectively reverses the decline of cell viability in AFB1-exposed group (Fig. 1A and 1B). Calcein-AM/PI staining revealed that AFB1 exposure significantly decreased cell survival rate, but Fer-1 co-treatment improved cell survival rate compared to the AFB1-exposed group (Fig. 1C and D). Since excessive ROS accumulation can lead to cell damage and oxidative stress, we measured ROS levels using DCFH-DA. The results showed that AFB1 exposure induced ROS accumulation, which was effectively attenuated by Fer-1 co-treatment (Fig. 1E and F). Both glutathione (**GSH**) content and the GSH/GSSG ratio were decreased in the AFB1 exposure group, while Fer-1 co-treatment increased these parameters compared to AFB1 exposure alone (Fig. 1G). AFB1 exposure elevated the malondialdehyde (**MDA**) content, but Fer-1 co-treatment decreased the MDA content (Fig. 1H). Meanwhile, the decreased activities of superoxide dismutase (**SOD**) and catalase (**CAT**) in AFB1 exposure group were rescued by Fer-1 co-treatment (Fig. 1I and J). Thus, these results showed that Fer-1 treatment could alleviate AFB1-induced excessive oxidative stress.

### AFB1 triggers ferroptosis in duck granulosa cells

**Fig. 2:**
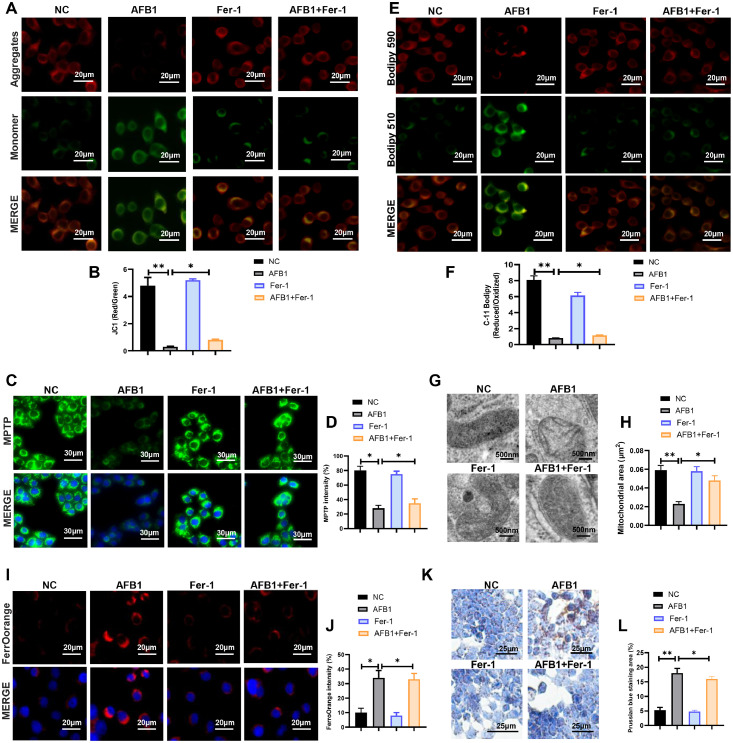
**AFB1 triggers ferroptosis in duck granulosa cells.** (A, B) Mitochondrial membrane potential and quantification of the JC-1 red/green fluorescence ratio in duck granulosa cells treated with AFB1 and Fer-1. (C, D) Lipid peroxidation analysis and quantification of C11-BODIPY 581/591 fluorescence in duck granulosa cells treated with AFB1 and Fer-1. Oxidized status (Green) and reduced status (Red) of C11-BODIPY 581/591. (E, F) Mitochondrial permeability transition pore (MPTP) and quantification of MPTP in duck granulosa cells treated with AFB1 and Fer-1. (G, H) Representative electron microscopic photographs and quantification of mitochondrial area in duck granulosa cells treated with AFB1 and Fer-1. (I, J) Representative micrographs and quantification analysis of FerroOrange staining in duck granulosa cells treated with AFB1 and Fer-1. (K, L) Representative micrographs and quantification analysis of Prussian blue staining in duck granulosa cells treated with AFB1 and Fer-1. All data are mean ± SD; *p < 0.05, **p < 0.01.

Excessive ROS production is a hallmark of mitochondrial dysfunction, thus we systematically evaluated mitochondrial structure and function. We first assessed the mitochondrial membrane potential and permeabilization (Fig. 2A-D). AFB1 exposure reduced membrane potential and increased membrane permeabilization, whereas these effects were markedly ameliorated by Fer-1 co-treatment (Fig. 2A-D). C11-BODIPY staining revealed that AFB1 exposure promoted lipid peroxidation, which was attenuated by Fer-1 co-treatment (Fig. 2E and F). Subsequently, we further analyzed the mitochondrial ultrastructure by transmission electron microscopy (TEM). Compared with the control group, we found that AFB1 exposure induced mitochondria shrunken and ruptured outer membrane, but Fer-1 co-treatment protected mitochondrial size and membrane integrity (Fig. 2G and 2H). Ferroptosis is characterized by lipid peroxidation and ROS accumulation, we hypothesized that AFB1 may induce ferroptosis. Confocal imaging showed that Fe^2+^ intensity was significantly greater in AFB1 exposure than in control group, and this increase could be mitigated by Fer-1 co-treatment (Fig. 2I and J). Meanwhile, Prussian blue staining also revealed massive iron deposition in AFB1 exposure, but Fer-1 co-treatment reduced this accumulation (Fig. 2K and L). In conclusion, these findings confirmed that Fer-1 treatment effectively mitigate AFB1-induced ferroptosis in duck granulosa cells.

### RNA-seq analysis of differentially expressed genes in AFB1-exposed duck granulosa cells

**Fig. 3:**
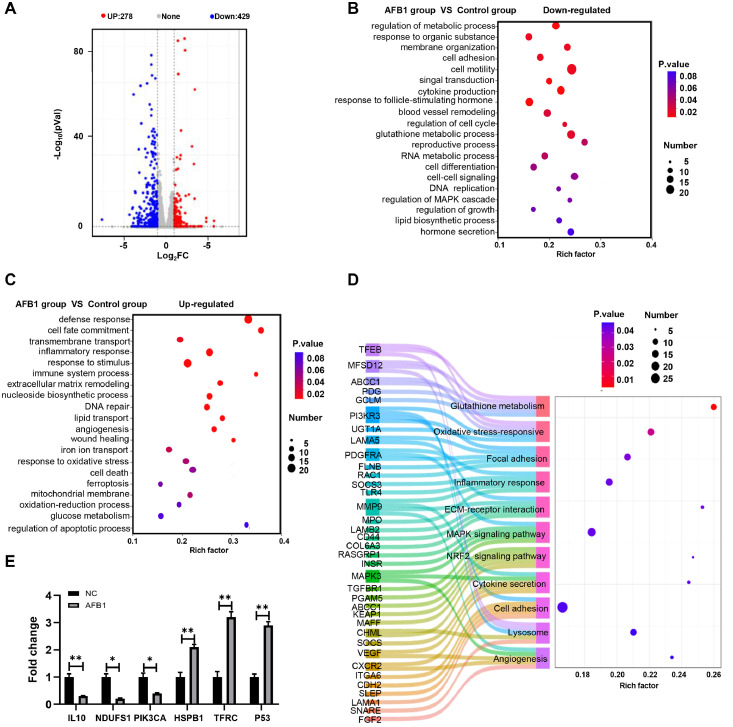
**Transcriptomic changes in AFB1-exposed duck granulosa cells. (**A) Volcano plot of differential gene expression analysis of duck granulosa cells between control and AFB1-exposed group. (n = 4). Differentially expressed genes (DEGs) were extracted with |log_2_Fc| > 1 and p-adj < 0.05. (B, C) GO analysis of downregulated (B) and upregulated (C) genes in AFB1-exposed group compared to control group. (D) KEGG pathway enrichment analysis of the DEGs between control and AFB1-exposed group. (E) Quantitative RT-PCR analysis of IL10, NDUFS1, PIK3CA, HSPB1, TFRC, P53 in duck granulosa cells between control and AFB1-exposed group. All data are mean ± SD; *p < 0.05, **p < 0.01.

To explore the mechanism by which AFB1 triggers oxidative stress and ferroptosis in duck granulosa cells, we employed transcriptome analysis of control and AFB1 exposure group using RNA-seq. There were 707 differentially expressed transcripts (|log_2_Fc| > 1 and *p*-*adj* < 0.05) with 278 transcripts up-regulated and 429 transcripts down-regulated in AFB1 exposure group compared to control group (Fig. 3A). GO analysis revealed that down-regulated genes in AFB1 exposure group were mostly enriched in terms related to “cytokine production”, “response to follicle-stimulating hormone”, “regulation of cell cycle”, “reproductive process”, and “hormone secretion” (Fig. 3B, Table S3). Up-regulated genes in the AFB1 exposure group were enriched in the processes of “defense response”, “iron ion transport”, “response to oxidative stress”, “ferroptosis” and “mitochondrial membrane” (Fig. 3C, Table S4). KEGG pathway analysis showed the differentially expressed genes involved in multiple signal transductions, including “oxidative stress-responsive pathway”, “NRF2 signaling pathway”, “MAPK signaling pathway”, and “ECM-receptor interaction pathway” (Fig. 3D). Meanwhile, the differentially expressed genes identified by RNA-seq were validated using quantitative real-time PCR (Fig. 3E). In summary, RNA-seq results demonstrated that AFB1 exposure affects the genes and pathways involved in oxidative stress and ferroptosis.

### AFB1 promotes ferroptosis by suppressing the NRF2 signaling pathway

**Fig. 4:**
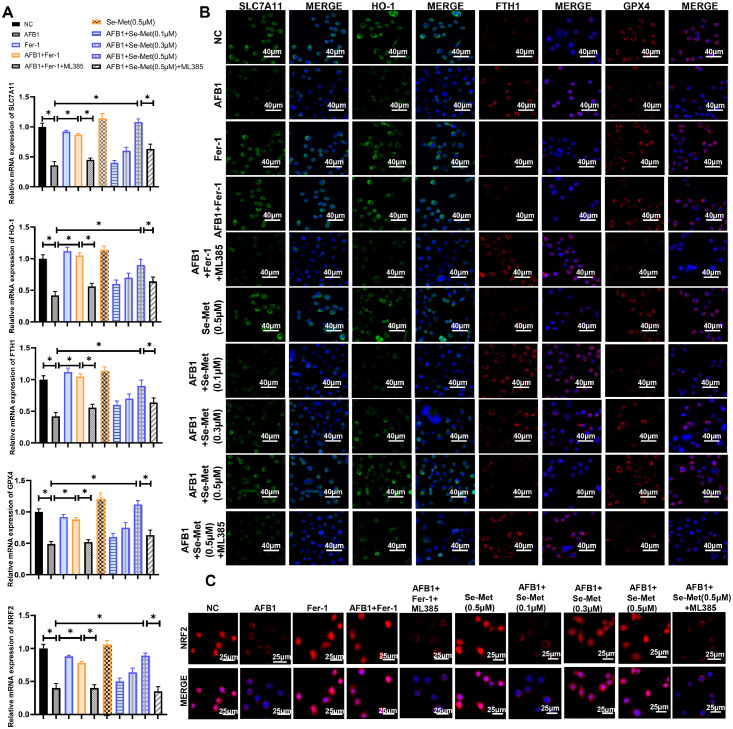
**AFB1 induces ferroptosis via the NRF2 signaling pathway in duck granulosa cells. (**A) Quantitative RT-PCR analysis of SLC7A11, HO-1, FTH1, GPX4, NRF2 in duck granulosa cells treated with AFB1, Fer-1, ML385, and different concentrations of Se-Met (0.1, 0.3 0.5 μM). (B) Immunofluorescence staining of SLC7A11, HO-1, FTH1, GPX4 in duck granulosa cells treated with AFB1, Fer-1, ML385, and different concentrations of Se-Met (0.1, 0.3 0.5 μM). (C) Immunofluorescence staining of NRF2 in duck granulosa cells treated with AFB1, Fer-1, ML385, and different concentrations of Se-Met (0.1, 0.3 0.5 μM). All data are mean ± SD; *p < 0.05, **p < 0.01.

Fer-1 co-treatment could reverse AFB1-induced ferroptosis in duck granulosa cells, and we next investigated the signaling pathway by which AFB1 regulates ferroptosis. Transcriptome analysis revealed that the NRF2 signaling pathway is involved in AFB1-induced ferroptosis and we assessed the expression of NRF2 signaling pathway-related genes (SLC7A11, HO-1, GPX4, NRF2, FTH1). qRT-PCR analysis showed the expression levels of SLC7A11, HO-1, GPX4, NRF2 were decreased in AFB1 exposure, whereas the expression of FTH1 was increased (Fig. 4A). Fer-1 co-treatment significantly ameliorated the aberrant expression of these NRF2 signaling pathway-related genes (Fig. 4A). Meanwhile, the immunofluorescence staining showed that these gene expression are consistent with the results of qRT-PCR (Fig. 4B). Because NRF2 is a cytosolic protein capable of nuclear translocation and transcriptional activation exposure to oxidative stress (Verma et al., 2015), we analyzed its expression distribution in nuclear and cytoplasmic components. Immunofluorescence showed that AFB1 exposure decreased the nuclear NRF2 accumulation compared with control group (Fig. 4C). However, Fer-1 co-treatment prevented the reduction of nuclear NRF2 level (Fig. 4C). Then we used the inhibitor of NRF2 (**ML385**) to further validate the impact of NRF2 signaling on AFB1-induced ferroptosis. The results showed that ML385 significantly attenuated both Fer-1-mediated restoration of NRF2 pathway gene expression (Fig. 4A and B) and NRF2 nuclear translocation (Fig. 4C). These observations indicated that AFB1-induced ferroptosis via NRF2 signaling pathway in duck granulosa cells.

### Se-met ameliorates AFB1-induced oxidative stress

**Fig. 5:**
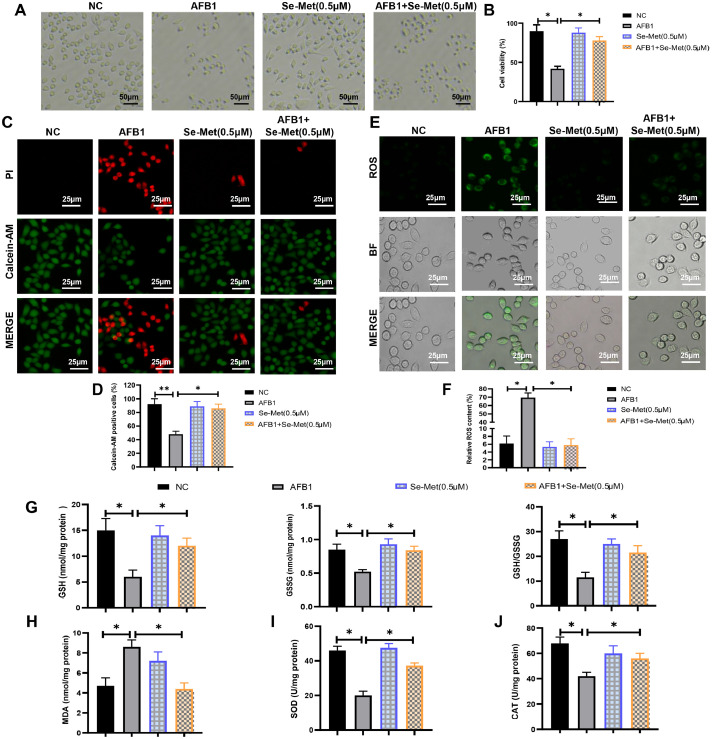
**Se-Met meliorates AFB1-induced oxidative stress in duck granulosa cells.** (A, B) Representative images and cell viability of duck granulosa cells treated with AFB1 and Se-Met. (C, D) Representative micrographs and quantification analysis of Calcein–AM/PI double staining in duck granulosa cells treated with AFB1 and Se-Met. (E, F) Fluorescence intensity and quantification analysis of ROS in duck granulosa cells treated with AFB1 and Se-Met. (G) The content of GSH, GSSG and the ratio of GSH/GSSG in duck granulosa cells treated with AFB1 and Se-Met. (H-J) The content of MDA (H), SOD (I), CAT (J) in duck granulosa cells treated with AFB1 and Fer-1. All data are mean ± SD; *p < 0.05, **p < 0.01.

Selenium (Se) has been known to play a pivotal role in mitigating oxidative stress and ferroptosis, and seleniummethionine (**Se-Met**) alleviates ferroptosis via the NRF2/GPX4 pathway in chicken and grass carp (Dong et al., 2024; Yu et al., 2024). Thus, we investigated whether Se-Met participates in regulating AFB1-induced ferroptosis in this study. First, we found that Se-Met co-treatment reversed the suppression of cell viability and cell growth in AFB1 exposure group (Fig. 5A-D). Furthermore, Se-Met effectively reduced ROS accumulation under AFB1 exposure (Fig. 5E and 5F). We also measured the levels of GSH, GSH, MDA, SOD, and CAT, and found that Se-Met co-treatment enhanced the antioxidant capacity compared with AFB1 exposure group (Fig. 5G-J). Taken together, these results demonstrate that Se-Met markedly attenuate AFB1-induced oxidative stress.

### Se-met alleviates AFB1-induced ferroptosis via NRF2 pathway activation

**Fig. 6:**
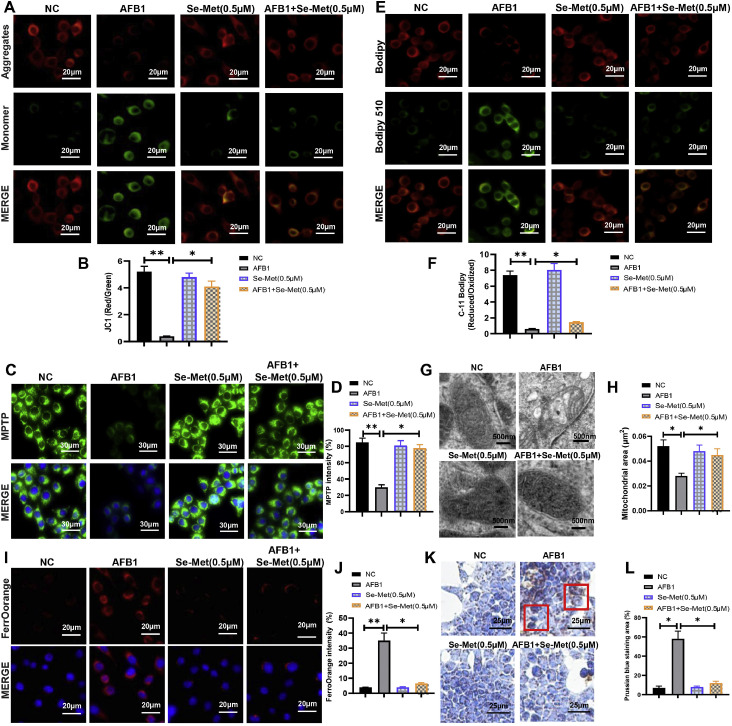
**Se-Met mitigates AFB1-induced ferroptosis in duck granulosa cells.** (A, B) Mitochondrial membrane potential and quantification of the JC-1 red/green fluorescence ratio in duck granulosa cells treated with AFB1 and Se-Met. (C, D) Mitochondrial permeability transition pore (MPTP) and quantification of MPTP in duck granulosa cells treated with AFB1 and Se-Met. (E, F) Lipid peroxidation analysis and quantification of C11-BODIPY 581/591 fluorescence in duck granulosa cells treated with AFB1 and Se-Met. Oxidized status (Green) and reduced status (Red) of C11-BODIPY 581/591. (G, H) Representative electron microscopic photographs and quantification of mitochondrial area in duck granulosa cells treated with AFB1 and Se-Met. (I, J) Representative micrographs and quantification analysis of FerroOrange staining in duck granulosa cells treated with AFB1 and Se-Met. (K, L) Representative micrographs and quantification analysis of Prussian blue staining in duck granulosa cells treated with AFB1 and Se-Met. All data are mean ± SD; *p < 0.05, **p < 0.01.

The mitochondrial membrane potential and permeabilization were improved in Se-Met co-treatment compared with AFB1 exposure group (Fig. 6A-D). And Se-Met co-treatment suppressed lipid peroxidation in AFB1 exposure (Fig. 6E and F). TEM showed that the mitochondria shrunken and ruptured outer membrane in AFB1 exposure were ameliorated by Se-Met treatment (Fig. 6G and H). Meanwhile, Se-Met co-treatment reduced iron deposition in AFB1 exposure (Fig. 6I-L). Moreover, Se-Met co-treatment rescued the AFB1-induced dysregulation of NRF2 pathway genes (HO-1, FTH1, GPX4, NRF2, SLC7A11) in a dose-dependent manner (Fig. 4A-C). Based on these results, we conclude that Se-Met could activate the NRF2 signaling pathway to resist AFB1-induced ferroptosis in duck granulosa cells.

### Se-met attenuates AFB1-induced ovarian toxicity in ducks

**Fig. 7:**
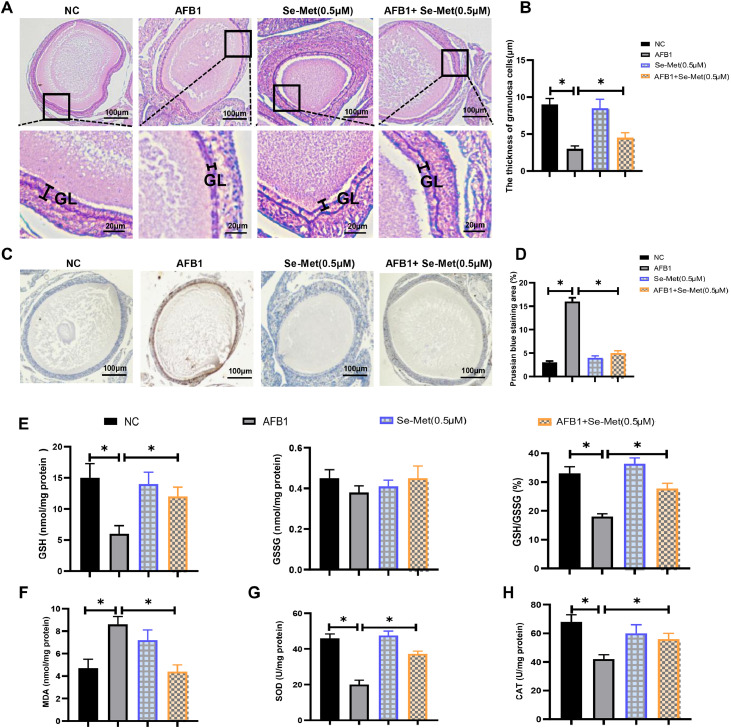
**Se-Met improved AFB1-induced ovarian dysfunction.** (A) H&E staining of ovarian sections in *vitro* cultured duck ovaries treated with AFB1 and Se-Met. GL: granulosa cell layer. (B) Thickness of granulosa cells layer in *vitro* cultured duck ovaries treated with AFB1 and Se-Met. (C, D) Representative micrographs and quantification analysis of Prussian blue staining of ovarian sections in *vitro* cultured duck ovaries treated with AFB1 and Se-Met. (E) The content of GSH, GSSG and the ratio of GSH/GSSG in *vitro* cultured duck ovaries treated with AFB1 and Se-Met. (F-H) The content of MDA (F), SOD (G), CAT (H) in *vitro* cultured duck ovaries treated with AFB1 and Se-Met. All data are mean ± SD; *p < 0.05, **p < 0.01.

Next, we explored the effects of AFB1 on follicular development and the protective role of Se-Met using in *vitro* cultured duck ovaries. Histological analysis revealed that AFB1 exposure significantly reduced granulosa cell layer thickness compared to control (Fig. 7A and B). Importantly, Se-Met effectively restored granulosa cell layer thickness, suggesting its protective role against AFB1-induced follicular developmental abnormalities (Fig. 7A and B). Prussian blue staining demonstrated iron deposition in AFB1-exposed ovaries, which was attenuated by Se-Met co-treatment (Fig. 7C and D). Moreover, evaluation of ovarian antioxidant capacity showed that Se-Met improved the reduced antioxidant activity in AFB1-exposed ovaries (Fig. 7E-H). Collectively, Se-Met ameliorates AFB1-induced ovarian dysfunction via maintaining follicular structure, reducing iron accumulation, and enhancing antioxidant capacity.

## Discussion

AFB1 is frequently found in food and feed that cannot be effectively eliminated through normal metabolic pathways (Yang et al., 2024a). With the accumulation of AFB1 in the body over time, AFB1 causes serious damage to multiple systems and organs, including the liver, kidney, heart, intestine, and endocrine system (Cheng et al., 2023c). Furthermore, AFB1 exposure induces significant reproductive toxicity, which severely affects the development of agriculture and animal husbandry. Hence, elucidating the molecular mechanisms of AFB1 intoxication and developing targeted therapeutic interventions are critical for preserving fertility. In this study, we investigated the toxicological mechanisms of AFB1 exposure in granulosa cells and explored the potential protective effect of Se-Met in ducks. These results demonstrated that AFB1 exposure induces excessive oxidative stress in duck granulosa cells, leading to mitochondrial dysfunction and ferroptosis. Meanwhile, we found that Se-Met could effectively alleviate AFB1-induced abnormal phenomenon via the NRF2 signaling pathway.

Mycotoxins exposure triggers oxidative stress and causes severe damage to multiple tissues and organs (Frangiamone et al., 2024). Oxidative stress reflects an imbalance between the production of free radicals and the antioxidant defense system, which is closely related to reproductive dysfunction (Showell et al., 2020). For instance, AFB1 induces excessive oxidative stress via PI3K/AKT signaling pathway, which suppresses oocytes maturation and blastocysts development in mice (Zhang et al., 2023). AFB1 triggers ROS accumulation, cell cycle arrest and apoptosis in porcine granulosa cells, and leads to ovarian dysfunction (Li et al., 2024). Moreover, NRF2-mediated antioxidant pathway has been shown to partially protect primordial germ cells from AFB1-induced oxidative stress and cell death (Niu et al., 2025). In this study, we observed that AFB1 induces oxidative stress in duck granulosa cells, characterized by excessive ROS accumulation and abnormal alterations in redox homeostasis markers, including the GSH/GSSG ratio, MDA levels, and the activities of SOD and CAT. Meanwhile, AFB1 exposure inhibits NRF2 signaling pathway, which is consistent with the previous studies. Collectively, these findings elucidated that AFB1 exposure induces oxidative stress and represses antioxidant pathways in duck granulosa cells.

Oxidative stress promotes lipid peroxidation and subsequently causes mitochondrial dysfunction (Zhang et al., 2021). Ferroptosis, a novel form of programmed cell death driven by iron-dependent lipid peroxidation, is primarily initiated by oxidative stress (Lin et al., 2025). Recent reports have shown that AFB1 exposure disrupts redox homeostasis and enhances lipid peroxidation, and then induces ferroptosis in the hippocampus (Yang et al., 2024b). Furthermore, AFB1 exposure triggers mitochondrial oxidative stress and ferroptosis, contributing to growth retardation and renal injury in ducks (Liu et al., 2023a). Indeed, the exhaustion of cellular antioxidants, suppression of enzymatic antioxidant defenses, accumulation of lipid peroxidation and iron are the hallmark events in ferroptosis (Noguchi et al., 2025). Importantly, these landmark changes of ferroptosis were all observed on AFB1-exposed duck granulosa cells in this study, and the ferroptosis inhibitor (Ferrostatin-1, **Fer-1**) alleviated these abnormalities. Additionally, the cystine/glutamate antiporter system (SLC7A11-SLC3A2) confers cellular resistance to ferroptosis by sustaining glutathione (GSH) biosynthesis (Jiang et al., 2024). As a key negative regulator of ferroptosis, GPX4 suppresses ferroptosis by reducing lipid hydroperoxides to non-toxic alcohols and attenuating ROS generation (Wang et al., 2023). Our results demonstrated that AFB1 exposure significantly downregulates the expression of GPX4 and SLC7A11, whereas Fer-1 effectively reverses this suppression. Overall, these results indicated that AFB1 induces ferroptosis by compromising the cellular antioxidant defense system.

As the master regulator of redox homeostasis, nuclear factor erythroid 2-related factor 2 (**NRF2**) exerts cytoprotective effects through nuclear translocation and transcriptional activation of downstream antioxidant genes, including glutathione peroxidase 4 (GPX4) and heme oxygenase-1 (HO-1) (Yang et al., 2024c). These effectors coordinately regulate multiple antioxidant defense systems, such as glutathione metabolism, iron deposition, lipid peroxidation, and mitochondrial quality. Notably, HO-1 serves as a critical component of antioxidant network by catalyzing the rate-limiting degradation of heme into ferrous iron, carbon monoxide, and biliverdin. For instance, Tagitinin C induces ferroptosis via ER stress-mediated activation of NRF2-HO-1 signaling pathway in colorectal cancer cells (Wei et al., 2021). Glutathione peroxidase 4 (GPX4) is a selenium-containing antioxidant enzyme, and plays a pivotal role in ferroptosis prevention by reducing lipid peroxidation. Recent studies demonstrated that PRDM16 suppresses ferroptosis to protect against acute kidney injury through the NRF2-GPX4 axis (Zheng et al., 2024). Moreover, activation of NRF2 and its nuclear translocation suppresses ferroptosis in ovarian granulosa cells, thereby ameliorating ovarian dysfunction (Dai et al., 2024; Xu et al., 2025). In our study, transcriptome sequencing revealed that AFB1 exposure affects mitochondrial function, cell cycle, ferroptosis, and antioxidant signaling pathways, especially the NRF2 pathway is significantly enriched. Meanwhile, AFB1 exposure disrupts the expression of NRF2 signaling pathway-related genes (SLC7A11, HO-1, GPX4, NRF2) and inhibits NRF2 nuclear translocation. Conversely, NRF2 inhibitor ML385 substantially suppressed NRF2 signaling pathway and blocked the anti-ferroptosis effect of Fer-1. Accordingly, AFB1 can trigger ferroptosis by suppressing NRF2 signaling pathway in duck granulosa cells.

Selenium (Se), an essential trace element vital for maintaining redox homeostasis in mammals, exerts its biological functions through selenoprotein families, particularly glutathione peroxidases (GPXs) (Qiao et al., 2022). Among various Se compounds, selenomethionine (Se-Met) represents an organic Se form characterized by high bioavailability, potent biological activity, and low toxicity (Bi et al., 2022). Studies have demonstrated that Se-Met exhibits protective effects against various diseases, including renal injury, brain damage, vascular disorders, and intestinal barrier dysfunction (Dong et al., 2024). These protective effects may be attributed to its antioxidant properties that effectively neutralize ROS and cytotoxic substances. For instance, Se-Met supplementation suppresses ER stress-induced ferroptosis by preserving GPX4 activity. Se-Met mitigates decabromodiphenyl-induced oxidative stress and ferroptosis through the NRF2-GPX4 axis in chicken brain (Zhu et al., 2023). Meanwhile, Se-Met reduces mitochondrial damage, enhances antioxidant enzyme activity, and suppresses ferroptosis through activation of the NRF2-HO-1 axis in chicken testicles (Li et al., 2025b). Se-Met reversed the dysregulated expression of NRF2 signaling pathway-related genes (SLC7A11, HO-1, GPX4, NRF2) and facilitates NRF2 nuclear translocation in this study, which displayed antioxidant and anti-ferroptotic functions. Therefore, our data revealed that Se-Met suppresses AFB1-induced ferroptosis by activating the NRF2 signaling pathway.

## Conclusion

**Fig. 8:**
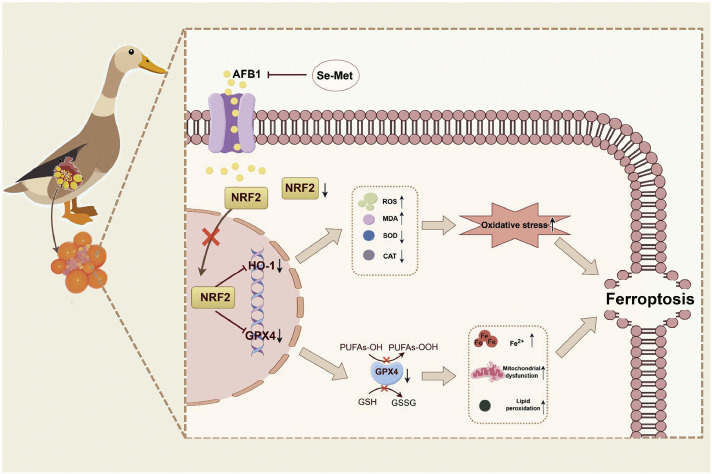
Schematic model depicting the molecular mechanism of Se-Met alleviating Aflatoxin B1 (AFB1)-induced ferroptosis through the NRF2 signaling pathway in duck granulosa cells.

In summary, this study elucidates the protective mechanism of Se-Met against AFB1-induced ferroptosis through the NRF2 signaling pathway in duck granulosa cells (Fig. 8). These findings provide novel insights into the molecular mechanisms of AFB1-induced ovarian dysfunction, and reveal a potential preventive and therapeutic strategy against AFB1-induced reproductive toxicity in poultry.

## Data availability

The data that support the findings of this study are available from the corresponding author upon reasonable request.

## CRediT authorship contribution statement

**Yaru Chen:** Writing – review & editing, Writing – original draft, Validation, Software, Formal analysis, Data curation. **Lei Wang:** Formal analysis, Conceptualization. **Ming Fu:** Resources, Methodology, Conceptualization. **Tao Huang:** Investigation. **Hao Zhang:** Methodology, Investigation. **Jie Shen:** Investigation. **Ailuan Pan:** Investigation. **Zhenhua Liang:** Methodology. **Jing Sun:** Investigation. **Jinping Du:** Investigation. **Jinsong Pi:** Resources, Methodology, Investigation, Conceptualization. **Yan Wu:** Writing – review & editing, Supervision, Resources, Project administration, Data curation, Conceptualization.

## Disclosures

The authors declare that they have no known competing financial interests or personal relationships that could have appeared to influence the work reported in this paper.

## Appendix Supplementary materials

Image, application 1

## Footnotes

Supplementary material associated with this article can be found, in the online version, at doi:10.1016/j.psj.2025.105993.
