# Supplementary material for: Aflatoxin B1 triggers ferroptosis via inhibiting the NRF2 signaling pathway in duck granulosa cells
Source: Poult Sci. 2025 Oct 24;104(12):105993. doi: 10.1016/j.psj.2025.105993 (PMC12639313; doi:10.1016/j.psj.2025.105993)
Supplement: Supplementary file 1 [file mmc1.doc]

**Supporting Information**

**Seleniummethionine alleviates Aflatoxin B1-induced** **ferroptosis by activating the NRF2 signaling pathway in duck granulosa cells**

Yaru Chen^1,2^, Wang Lei^3^, Ming Fu^1,2^, Tao Huang^1,2^, Hao Zhang^1,2^, Jie Shen^1,2^, Ailuan Pan^1,2^, Zhenhua Liang^1,2^, Jing Sun^1,2^and Jinping Du^1,2^, Yan Wu^1,2*^, Jinsong Pi^1,2*^

^1^ Institute of Animal Science and Veterinary Medicine, Hubei Academy of Agricultural Sciences, Wuhan 430064, China;

^2^ Hubei Key Laboratory of Animal Embryo Engineering and Molecular Breeding, Wuhan 430064, China;

^3^ Wuhan Green Giant Agriculture & Animal Husbandry Company Limited, Wuhan 432200, China

^*^ Correspondence and reprint requests to:

**Yan Wu;**

^*^Email: wuyanwh202112@hbaas.com;

**Jinsong Pi**;

^*^Email: [pijinsong@sina.com](mailto:pijinsong@sina.com).

Table of Contents (Supplementary Information)

Table S1: The sequences of primers used in this study;

Table S2: The information of antibodies in the study;

Table S3: Gene ontology (GO) analysis of down-regulated DEGs in AFB1-exposed group compared to control group;

Table S4: Gene ontology (GO) analysis of up-regulated DEGs in AFB1-exposed group compared to control group.

**Table S1:** The sequences of primers used in this study

| Genes | | Primer sequences（5’ to 3’） |
| --- | --- | --- |
| β-actin | F: CCGCTCTATGAAGGCTACGC  R: CTCTCGGCTGTGGTGGTGAA | |
| SLC7A11 | F: AGGGTCTGGTTGGCTTTTGT  R: TCCAGCCAAAGTCACCGTTA | |
| HO-1 | F: GCCTGGTTCAAGATACTACCTCT  R: TAAATCCCCACTGCCACGGT | |
| FTH1 | F: CACTTCCAGTTTTTGCCGGG  R: GGGAATGGCTGGTGCTCAAT | |
| GPX4 | F: CCAGCCCCTACACCTACCTC  R: GCTGGGCTTGTCAGTTTCTC | |
| NRF2 | F: CCGGAGAGGAGATTTGAGTG  R: TGAGGTCCAGGGTTTCTGAC | |

**Table S2:** The information of antibodies in the study

| Antibody | | Manufacturer | | Product No. | | Dilution ration |
| --- | --- | --- | --- | --- | --- | --- |
| ACTIN | ABclonal | | AC038 | | 1:100 | |
| SLC7A11 | Sigma | | SAB2500951 | | 1:50 | |
| HO-1 | Abclonal | | A1346 | | 1:50 | |
| FTH1 | Proteintech | | 11682-1-AP | | 1:100 | |
| GPX4 | Abclonal | | A11243 | | 1:100 | |
| NRF2 | Abclonal | | A0674 | | 1:200 | |
| FITC Goat Anti-Rabbit IgG (H+L) | Sigma | | F0257 | | 1:200 | |
| Cy3 Goat Anti-Rabbit IgG (H+L) | Proteintech | | SA00009-1 | | 1:100 | |

**Table S3: Gene ontology (GO) analysis of down-regulated DEGs in AFB1-exposed group compared to control group.** The down-regulated DEGs (log_2_Fc ＜1 and *p* < 0.05) were subjected to GO analyses by DAVID (https://david.ncifcrf.gov/)

| GO_Term | | GO_function | | S gene number | | TS gene number | | B gene number | | TB gene number | | *p* value |
| --- | --- | --- | --- | --- | --- | --- | --- | --- | --- | --- | --- | --- |
| regulation of metabolic process | Biological Process | | 13 | | 1028 | | 156 | | 21017 | | 0.00445 | |
| response to organic substance | Biological Process | | 11 | | 1028 | | 141 | | 21017 | | 0.01023 | |
| membrane organization | Biological Process | | 11 | | 1028 | | 124 | | 21017 | | 0.01126 | |
| cell adhesion | Biological Process | | 10 | | 1028 | | 103 | | 21017 | | 0.01276 | |
| cell motility | Biological Process | | 20 | | 1028 | | 248 | | 21017 | | 0.01488 | |
| singal transduction | Biological Process | | 9 | | 1028 | | 94 | | 21017 | | 0.01697 | |
| cytokine production | Biological Process | | 12 | | 1028 | | 138 | | 21017 | | 0.01704 | |
| response to follicle-stimulating hormone | Biological Process | | 11 | | 1028 | | 125 | | 21017 | | 0.01888 | |
| blood vessel remodeling | Biological Process | | 11 | | 1028 | | 113 | | 21017 | | 0.02069 | |
| regulation of cell cycle | Biological Process | | 6 | | 1028 | | 87 | | 21017 | | 0.01685 | |
| glutathione metabolic process | Biological Process | | 12 | | 1028 | | 135 | | 21017 | | 0.01836 | |
| reproductive process | Biological Process | | 10 | | 1028 | | 104 | | 21017 | | 0.02164 | |
| RNA metabolic process | Biological Process | | 11 | | 1028 | | 112 | | 21017 | | 0.03546 | |
| cell differentiation | Biological Process | | 11 | | 1028 | | 115 | | 21017 | | 0.02487 | |
| cell-cell signaling | Biological Process | | 11 | | 1028 | | 117 | | 21017 | | 0.03489 | |
| DNA replication | Biological Process | | 7 | | 1028 | | 89 | | 21017 | | 0.04219 | |
| regulation of MAPK cascade | Biological Process | | 6 | | 1028 | | 72 | | 21017 | | 0.05015 | |
| regulation of growth | Biological Process | | 6 | | 1028 | | 76 | | 21017 | | 0.05418 | |
| lipid biosynthetic process | Biological Process | | 10 | | 1028 | | 64 | | 21017 | | 0.06147 | |
| hormone secretion | Biological Process | | 11 | | 1028 | | 119 | | 21017 | | 0.07549 | |

**Table S4: Gene ontology (GO) analysis of up-regulated DEGs in AFB1-exposed group compared to control group.** The up-regulated DEGs (log_2_Fc > 1 and *p* < 0.05) were subjected to GO analyses by DAVID (https://david.ncifcrf.gov/)

| GO_Term | | GO_function | | S gene number | | TS gene number | | B gene number | | TB gene number | | *p* value |
| --- | --- | --- | --- | --- | --- | --- | --- | --- | --- | --- | --- | --- |
| defense response | Biological Process | | 22 | | 1028 | | 256 | | 21017 | | 0.01328 | |
| cell fate commitment | Biological Process | | 14 | | 1028 | | 154 | | 21017 | | 0.01439 | |
| transmembrane transport | Biological Process | | 11 | | 1028 | | 132 | | 21017 | | 0.01547 | |
| inflammatory response | Biological Process | | 13 | | 1028 | | 146 | | 21017 | | 0.01638 | |
| response to stimulus | Biological Process | | 16 | | 1028 | | 187 | | 21017 | | 0.01529 | |
| immune system process | Biological Process | | 7 | | 1028 | | 98 | | 21017 | | 0.01699 | |
| extracellular matrix remodeling | Biological Process | | 11 | | 1028 | | 129 | | 21017 | | 0.01412 | |
| nucleoside biosynthetic process | Biological Process | | 12 | | 1028 | | 137 | | 21017 | | 0.01325 | |
| DNA repair | Biological Process | | 13 | | 1028 | | 152 | | 21017 | | 0.01754 | |
| lipid transport | Biological Process | | 13 | | 1028 | | 148 | | 21017 | | 0.01826 | |
| angiogenesis | Biological Process | | 12 | | 1028 | | 131 | | 21017 | | 0.01604 | |
| wound healing | Biological Process | | 8 | | 1028 | | 106 | | 21017 | | 0.02485 | |
| iron ion transport | Biological Process | | 11 | | 1028 | | 124 | | 21017 | | 0.03547 | |
| response to oxidative stress | Biological Process | | 10 | | 1028 | | 119 | | 21017 | | 0.06015 | |
| cell death | Biological Process | | 12 | | 1028 | | 128 | | 21017 | | 0.06946 | |
| ferroptosis | Biological Process | | 8 | | 1028 | | 107 | | 21017 | | 0.072410 | |
| mitochondrial membrane | Biological Process | | 10 | | 1028 | | 120 | | 21017 | | 0.06417 | |
| oxidation-reduction process | Biological Process | | 7 | | 1028 | | 87 | | 21017 | | 0.07784 | |
| glucose metabolism | Biological Process | | 9 | | 1028 | | 104 | | 21017 | | 0.06452 | |
| regulation of apoptotic process | Biological Process | | 8 | | 1028 | | 96 | | 21017 | | 0.08018 | |
